# Publishing and sharing multi-dimensional image data with OMERO

**DOI:** 10.1007/s00335-015-9587-6

**Published:** 2015-07-30

**Authors:** Jean-Marie Burel, Sébastien Besson, Colin Blackburn, Mark Carroll, Richard K. Ferguson, Helen Flynn, Kenneth Gillen, Roger Leigh, Simon Li, Dominik Lindner, Melissa Linkert, William J. Moore, Balaji Ramalingam, Emil Rozbicki, Aleksandra Tarkowska, Petr Walczysko, Chris Allan, Josh Moore, Jason R. Swedlow

**Affiliations:** Centre for Gene Regulation & Expression, University of Dundee, Dundee, Scotland UK; Glencoe Software, Inc., Seattle, WA USA

## Abstract

Imaging data are used in the life and biomedical sciences to measure the molecular and structural composition and dynamics of cells, tissues, and organisms. Datasets range in size from megabytes to terabytes and usually contain a combination of binary pixel data and metadata that describe the acquisition process and any derived results. The OMERO image data management platform allows users to securely share image datasets according to specific permissions levels: data can be held privately, shared with a set of colleagues, or made available via a public URL. Users control access by assigning data to specific Groups with defined membership and access rights. OMERO’s Permission system supports simple data sharing in a lab, collaborative data analysis, and even teaching environments. OMERO software is open source and released by the OME Consortium at www.openmicroscopy.org.

## Introduction

Imaging has always been a critical technology for the fields of cell and developmental biology. Results from microscopy were initially recorded by drawing, and then photography, with the goal of documenting, transmitting, and communicating results viewed by a scientist to other colleagues. These media were historically used to record the stages of embryonic development and the localization of gene expression patterns throughout the developing embryo. The advent of digital imaging, combined with confocal (White et al. 1987), two photon (Denk et al. 1990), and most recently light sheet microscopy (Huisken et al. 2004), has enabled a revolution in the study of embryology, development, and biology in general. These advances are now well established, but it is important to consider that they are accompanied by significant steps in automation associated with imaging devices (e.g., robots, multi-well plate holders, etc.), increasing detector sensitivity, and substantial changes in detector area and the number of pixels in each recorded image.

These advances in automation and photodetection are now used in experiments with large numbers of mutant or other genetic perturbations, to follow the molecular and structural processes that define organism development at scale, often across long timescales. The datasets generated in these experiments are multi-dimensional [encompassing space, time, and molecular composition (Andrews et al. 2002)], heterogeneous (containing many different forms of image data and metadata), and complex (not conforming to simple, defined standards). Data size varies, but datasets in the multi-GB to TB range are now routine, so laboratories and institutions using these new imaging modalities have emerged as enterprise data generators—by no means the aim of their experimental goals, but a necessary consequence of the scale of the projects these labs undertake. Laboratories and collaborations then require data management solutions to provide access to large, heterogeneous, complex datasets, manage analysis of the data, and share data among collaborators, and to publish data for access and re-analysis by the worldwide community.

In this article, we review the use of OMERO, an open source image software platform designed for these purposes and focus in particular on the methods OMERO uses to control and allow access to data between collaborators and to publish data on-line for public access, and how OMERO integrates into on-line publication systems.

### The problem—integrity, reuse, and resources

The evolution of imaging in cell and developmental biology is part of a larger trend occurring throughout the biological sciences towards making scientific datasets generated from public- or charity-funded projects publicly available for review and re-analysis. In particular, this involves making data associated with a published study available alongside the publication. Making raw and processed data available ensures a degree of integrity that is critical for the continued support of the scientific community by the public and provides opportunities for reusing data for aims that were not included in the original study. In the case of modern embryology, time lapse multi-dimensional images of developing embryos serve as benchmarks for algorithm developers building the next generation of, for example, object identification and tracking tools that produce maps of cell movements and interactions. They are also potential resources for studies comparing the relationship between evolution and development. Finally, at least in principle, these datasets may be resources for the community and if recorded, analyzed and annotated using standardized protocols, can be usefully shared with the wider community.

We are several years from achieving this vision, but the pace of the development of imaging technology—and in particular the increasing scale of datasets acquired in modern imaging systems—demands that the community begins building, deploying, and using modern data management strategies. The wider biological community will need to build skills and expertise in data management and curation, and the community will need data management, sharing, and publications tools capable of handling the data volumes associated with modern biology.

### OMERO summary

The Open Microscopy Environment (OME) has been releasing the OME Remote Objects (OMERO) software platform since 2007. OMERO is an enterprise data management application that combines mechanisms for storing and accessing image metadata, binary pixel data, annotations, and analytic output. OMERO is built as a client–server application that enables remote access to the data it holds. OMERO’s Permissions system controls access to that data ensuring that each dataset, image, annotation, or analytic result is only retrievable by those with correct permissions to do so. The server includes a Python-based scripting system for running processing and analysis tools and provides interfaces for data processing.

OMERO’s application programming interface (API) supports many different client applications cast in any modern processing environment (e.g., Java, C++, Python, and Matlab) and provides a flexible platform for storing, analyzing, and sharing large image datasets. Several examples of integrating analysis tools into OMERO have been published (Cuccuru et al. 2014; Cho et al. 2012; Devès et al. 2015; Warren et al. 2013; Swedlow et al. 2009; Leo et al. 2014).

A user establishes a connection with an OMERO system by logging in using SSL-encrypted authentication (Allan et al. 2012). Once logged in, the user interacts with two types of data: images, annotations, and results that she/he owns, or similar data owned by other users. The OMERO Permissions system defines the rights a user has for each element of data stored in an OMERO system. In its default configuration, this system allows a user full privileges and access to data she/he owns; data can be viewed, annotated, analyzed, or deleted. The OMERO Permissions system also defines the levels of access to data owned by other users, and controls whether data are visible only to users that are logged into the system, or are publically available to the outside world. In this paper, we focus on the principles and techniques that OMERO uses to control data access and that make OMERO an enterprise application for data sharing for collaborative work and data publication.

## Access control via OMERO groups

OMERO’s Permission system uses the concept of a *Group* as the basic mechanism for assigning access rights to data. In OMERO, access to any image, or its associated metadata, is set by the Group they are placed in. The system is described by three basic principles:

- Access to a specific image is changed by placing the image in a group with different permissions. In this way, OMERO separates the permissions and access state of the data from the data itself.
- A user always has unlimited access to his or her data, regardless of the permissions on the Group that holds the data. Permissions only specify how Group members can access and use data owned by other members of a Group.
- There is no limit to the number of Groups a user can join and no links between data in different Groups.

In constructing the OMERO Permissions system, we considered the possibility that, within a Group, it might be necessary to assign different rights to different users. We therefore provide three roles: an Administrator, a Group Owner, and a Group Member. A single Group can have any number of Users with any of these roles.

### Group roles

An Administrator is similar to a “root” user in Linux or OS X systems, or to an Administrator in Windows systems, and has the most liberal access rights to data in OMERO (see Table 1). For this reason, Administrators are normally encouraged not to execute non-systems actions, as there is risk of data modification or loss.

**Table 1:** Control of data access using the OMERO Permissions system

User role	Group Member				Group Owner				Administrator			
Group type	Private	Read-Only	Read-Annotate	Read-Write	Private	Read-Only	Read-Annotate	Read-Write	Private	Read-Only	Read-Annotate	Read-Write
Actions allowed on other users’ data												
View		*	*	*	*	*	*	*	*	*	*	*
Annotate			*	*		*	*	*		*	*	*
Delete				*	*	*	*	*	*	*	*	*
Edit				*	*	*	*	*	*	*	*	*
Move									*	*	*	*

Table shows the allowed actions for different user roles in an OMERO system. Users always have full access to their own data; access by other Users is defined by placing data in Groups with different Permission levels. Table shows the actions users can perform (*) on data owned by another user within a Group. User Action definitions: View, view another User’s images and regions of interest (ROIs); Annotate, add annotations (rating, tags, attachments, comments, ROIs) to another user’s data; Delete, remove another user’s images, and associated metadata from the OMERO system; Edit, change image names and descriptions; Move, copy, or move another user’s data from one group to another

Users must be members of at least one Group and a default group—the one they are operating in each time they log in—is assigned when their account is created. They will also be classified as a Group Owner or a Group Member.

In general, a Group Owner has a higher level of access than a Group Member, so that, for example, a project or group leader can exercise certain rights over the Group Members (e.g., members of his/her lab). Any number of users can be assigned to a Group (from 0 to the number of users on the system), and each of these users can be set as a Group Owner or Group Member in as many Groups as necessary. The use of these two permission levels enables some flexibility within the Permissions system, and means that within a Group, there can be users with different access rights and roles. As described below, the differences between Group Owners and Group Members depend on the Permissions level of the Group.

Once data are placed within a Group, it assumes the permissions and access rights assigned to that group. Every data element stored in the OMERO database contains a reference to the User that owns the data and the Group that holds the data, so access to any data element is explicitly stated at the level of that element.

### Group levels

Each Group in an OMERO system is assigned a specific Permissions level that defines how any member of the group can access data owned by other members. Available Permissions levels are Private, Read-Only, and Read-Annotate, and as of June 2015, Read-Write.

A Private Group is the most restrictive group. Group Members have no access to each other’s data, but Group Owners can view other Group Members’ data. This allows, for example, a PI access to data for the students in his/her group, but prevents anyone else in the group viewing each other’s data.

A Read-Only Group allows users to view each other’s data, but blocks Group Members from annotating each other’s data. Group Owners can view other Group Members’ data, and apply a subset of possible annotations. This allows Group Members to collaborate by viewing and interpreting data, but without making significant data additions or modifications. Following several requests from the community, Group Owners are allowed to read data from other group members and define regions of interest (ROIs) in another Group Member’s data. Note that the ROIs are always owned by the user who created them. This is an example of using Group Owners as data analysts who can access a Group’s data and perform analysis on it. In the future, we may add a new role to allow data analysis by Users without having to assign them the full rights of a Group Owner.

A Read-Annotate Group is a less restrictive permissions level and allows Group Members to view, annotate, and analyze each other’s data. It is designed to enable truly collaborative working between scientists, data analysts etc.

A Read-Write Group gives the freest access and permissions for users in OMERO. In addition to the collaborative annotation and analysis enabled in a Read-Annotate Group, the Read-Write permissions level allows modifications to data by Group Members, including editing and deletion. This unlimited access means that Group Members have the same access rights as the User who owns the data. We have only implemented this Permission level recently, in response to specific community requests. This type of Group should only be used where completely open, unfettered access to data is necessary for an effective collaboration.

A summary of different types of Users and their rights in the OMERO Permissions system is shown in Table 1.

## Managing OMERO groups

The OMERO Permissions system was designed assuming that Groups will consist of Users who are colleagues, or are somehow related to each other, and that permissions will be chosen based on how they work together in general rather than on an image-by-image basis. Users can place data in different Groups, with different Permissions levels and containing different Group Members and Owners to allow access and sharing of different datasets. Users and Groups can be created and managed by an Administrator, or can be created automatically and managed through an institutional LDAP directory.

This system of Group-based permissions ensures that secure, defined data access is easily implemented and maintained. Simply by importing new data into an existing Group, a User declares all the necessary permissions and access controls for that data. Each Group can be thought of as a secure, protected collection of data.

In all four types of Groups, there may be cases where a specific Group’s permissions must be modified after members have started adding and sharing data. The OMERO Administrator or a Group Owner can change the permissions level of a Group subject to certain limitations. A Private Group can raise its access level to a Read-Only or Read-Annotate Group, but it is not generally possible to reduce Permissions from Read-Only or Read-Annotate to Private. This prevents annotations or analysis from Group Members or Group Owners becoming orphaned during a change in Permissions. By contrast, it is possible to freely switch Permissions level in a Group between Read-Only, Read Annotate or Read-Write. Group Owners can also manage their Groups, including adding existing users and changing permission levels. Group Members cannot restrict access to their data even in a Private Group, to prevent data being hidden from authorized people in a system designed to facilitate sharing e.g., a Postdoc hiding data from a PI.

Further, changing the permissions on an individual image involves either changing the permissions of the Group containing the data or moving that image to a different Group with the required permissions. Placing data in two different Groups enables two different sharing policies, for example with different sets of colleagues. Currently, copying data to a new Group requires a physical copy of the data. While this is a less common use case, we are working towards reducing the duplication of multiple copies of data in future versions of OMERO.

## Use cases

Sharing data is a fundamental activity in science and all scientists share data with colleagues, collaborators, and publications. OMERO’s Permissions system was designed to be as generic, flexible, and support as many different sharing scenarios as possible. In our own implementations, we have developed several specific use cases for OMERO’s Permissions that serve as representative examples of different ways OMERO can be used:

- Secure, Confidential Data Access: In cases where OMERO holds confidential data, a Private Group ensures that Group Users cannot observe each other’s work. A Group Owner, which can be a person, or separate application with appropriate authentication, can connect to OMERO over a secure connection and deposit datasets in the Private Group, with ownership assigned to a Group User. All sessions, edits, annotations, or processing of the data by the Group User are logged in the OMERO Events table and are also visible to the Group Owner. A Group Owner, in this case, a trusted authority, can modify or delete any of the data accessed or generated by the Group User.
- Facility Manager imports: Imaging facilities are often run by Facility Managers. When guests visit a facility or data have to be moved from facility storage to central resources, Managers sometimes find themselves having to handle data for their users. If a Facility Manager is a Group Owner, she/he can import data as any other Group User, making the data available to the user for further examination and processing. We may also introduce a new ‘importer’ role in the future, separating this ability from the enhanced permissions available to Group Owners.
- Classroom Setting: Images that are part of a Read-Only group can be used as didactic tools in an educational environment. Students can browse, search, and view images and metadata on mobile devices and laptops. In situations where students are creating content independently, e.g., in a test or an assigned project, a Private group can be used where the instructor is a Group Owner in order to see all the data generated by the student Group Members. In an exam, students can be asked to draw regions of interest in response to exam questions. The instructor, through his/her rights as a Group Owner, can then review and evaluate the work.
- Collaborative Data Analysis: In Read-Only Groups, Group Owners can annotate and analyze other User’s data. In the future, in response to several requests, we plan to implement a Read-Analyze permissions level, to explicitly support collaborative data analysis.
- Paper preparation: for a lab actively working on a publication together, a Read-Write group may be more appropriate. This would allow each lab member to change dataset names, re-arrange images, and otherwise modify content as datasets and figures are refined.
- Paper publication: after a paper has been published, data associated with the paper can be moved to a special “publication” group with Read-Only access, ensuring datasets associated with public work are retained. In future versions of OMERO, we will introduce a truly Read-Only permission level to allow published data to be locked against any changes by any users, including the data owner.

## Publishing data with OMERO

OMERO provides a service for publishing data and enabling on-line access to the datasets it holds. This allows a lab, department, facility, or institution to publish specific datasets for public access, and thereby meet requirements for data access that are now emerging from funders and publishers. Enabling data publication requires specific configuration of the OMERO server that can only be performed by an Administrator. If these changes to the default configuration are made, then datasets become available via a standardized URL that can be embedded in web pages or other applications.

### OMERO configuration

OMERO uses the concept of a Public User to mediate the publication of data from any Group. A Public User added to any group makes the data in the Group publicly available, i.e., viewable from a URL. An Administrator or Group Owner is required to add the Public User to a Group rather than this being enabled by default; therefore this mechanism of publishing data cannot be activated by Members without elevated permissions. This protects data on an OMERO system from actively being published unless specifically designated to do so.

In practice, a Public User is a normal user that automatically logs into OMERO with an anonymous user id. This can be thought of as an extra level to OMERO’s authentication system, implemented at the client level. It is a powerful concept but should be used with caution, as merely adding a Public User to a Group makes all data contained within that group public.

### Handling data in a group with a public user

Once a Group is made “public” by the addition of a Public User then data in that Group are publicly available. Users can move data from other Groups into the Public Group, or indeed move data out that they prefer not to be published.

Image datasets in OMERO have several types of metadata associated with them. By default, the only metadata published alongside image data are the acquisition metadata and any associated regions of interest (ROIs). All other annotations, associated results, attached documents, etc. are protected from publication in OMERO in the default settings. Additional metadata can be published, if the server is configured to do so.

Regardless of whether a Group is public or not, the assigned permissions level is still operative. For this reason, most uses of data publication require Read-Only permissions on a Public Group. For example, for a Read-Only published Group, the owner of data in a published Group still can modify default rendering settings, annotations, etc. once data are published. Public Users can adjust rendering settings for display, but cannot save them or make them defaults for all other users.

### Accessing published data

Once data are published in OMERO, it is available for access, e.g., through a URL. Currently, OMERO does not provide a fully developed presentation of published data (e.g., a specifically designed web page for displaying published datasets). However, the data published in OMERO can be integrated into any other web page, and many of the functions available in the OMERO system are available through URLs. For example

Display a JPEG thumbnail of an image (where the ID of the image on this OMERO server is 3925540): https://www.nightshade.openmicroscopy.org/webgateway/render_thumbnail/3925540/

Display a full-resolution JPEG at a specific optical section (in this case, *z* = 3) and time point (*t* = 5): https://www.nightshade.openmicroscopy.org/webgateway/render_image/3925540/3/5/

Display a multi-dimensional viewer for viewing a 5D image: https://www.nightshade.openmicroscopy.org/webgateway/img_detail/3925540/

**Fig. 1:**
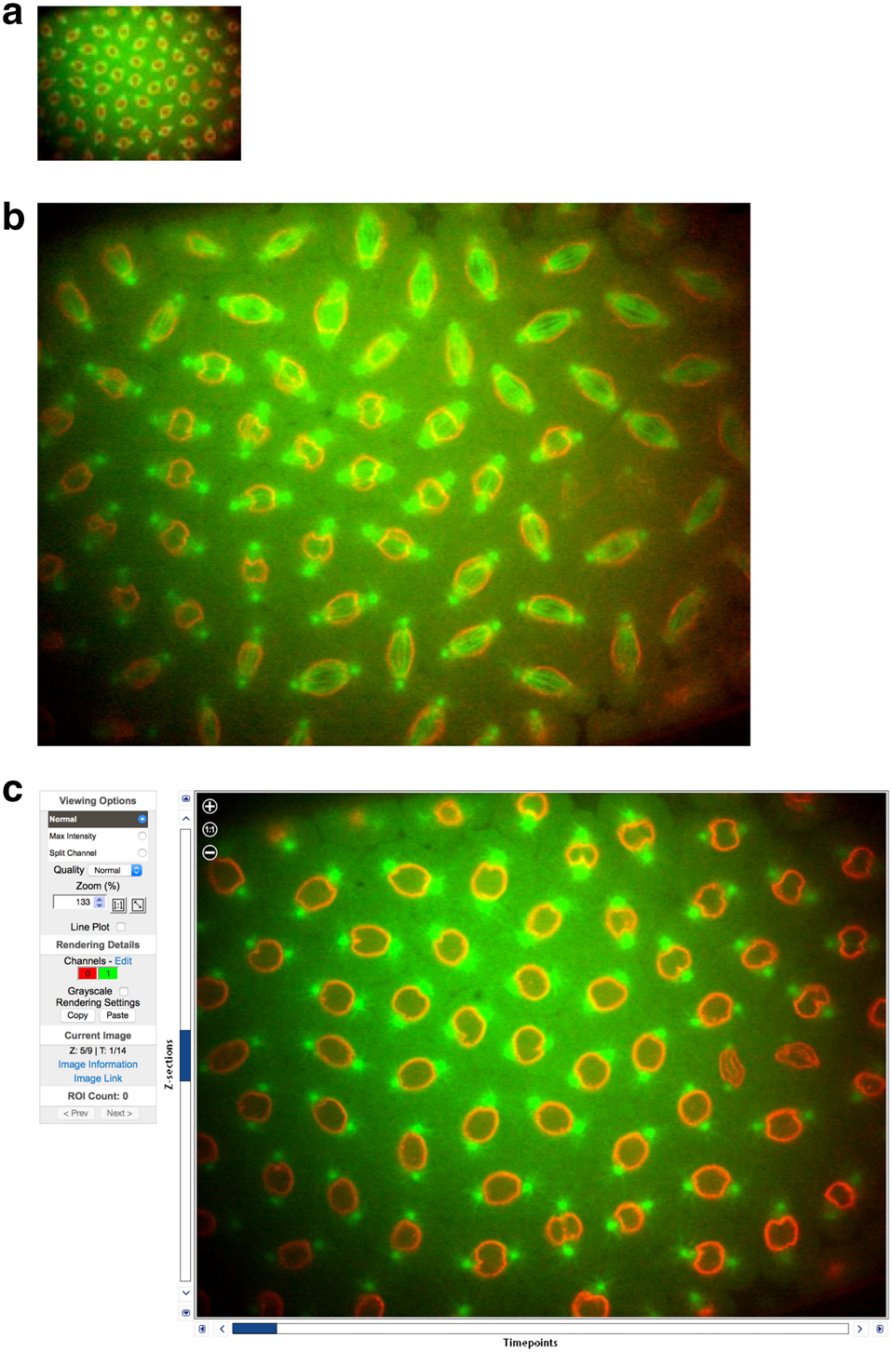
Publishing Image Data On-Line with OMERO. Results of the URLs shown in the text are shown. Each picture is a screenshot from a web browser, showing the JPEG generated from an OMERO server containing data in an original acquisition format. Original data published with (Civelekoglu-Scholey et al. 2010). **a** A JPEG image thumbnail returned by an OMERO server. **b** A full-size JPEG image returned from the server, rendering two fluorescent channels at a specified optical section and timepoint. **c** A full interactive multi-dimensional web browser-based viewer capable of displaying multi-GB scale datasets

The results of these URLS are shown in Fig. 1. These URLs access the OMERO WebGateway, which provides substantial image viewing and manipulation functionality, e.g., multi-dimensional visualization, image sizing, render settings, etc. Full on-line documentation of this resource is available.

## Next steps: data search and integration

With a solution for data publishing available from individual OMERO systems, it next becomes possible to consider linking data from several systems using a central broker or repository. It is certainly possible to record published images in central repositories—several are now available (Hill 2008; Lagerstedt et al. 2013; Kyoda et al. 2015; Neumann et al. 2010; Thompson et al. 2014), and more will come on-line over the next few years. As these resources propagate, it is essential to make them discoverable from either defined central resources (e.g., PubMed, ENSEMBL) or even search engines like Google, Yahoo, etc. This can be achieved by making standardized metadata presentations available to these resources. There are currently several efforts to present these metadata, either in public (e.g., Data Dryad) or publisher-based (e.g., Scientific Data or GigaScience) repositories.

A deeper challenge is the integration of separate resources to aggregate phenotypic data from different assays on, for example, the same or orthologous genes. Access to metadata that describe and define images—text-based annotations, ideally specific, community-agreed ontology-based phenotypic annotations—is the first step to integrating these data (Deans et al. 2015). The first repositories that aggregate image-based phenotypic screens are now becoming available (Krupke et al. 2008; Kirsanova et al. 2015), with limited examples of coherent, standardized, ontology-based annotation schemes. Going forward, we will require consistent ontological annotation of methods, imaging technology and detected phenotypes, and storage and access to these annotations through a defined interface. If this was achieved, then images recording and measuring phenotypes in distributed resources based on OMERO or other software technologies [e.g., Bisque (Kvilekval et al. 2010)] could be made discoverable by a central registry. It is most likely that the next practical step in the development of this technology will come in the integration of data from sites performing closely related work, e.g., phenotypic screening of a broad range of developmental processes in a single organism (Morgan et al. 2012). A major goal for OMERO development in the near term is the explicit support for storing and retrieving ontologies on the server and presenting these terms through the OMERO API to clients for annotation and search.

## Summary

Data sharing and publication are fundamental activities for any scientist. The size, complexity, and heterogeneity of imaging data, along with the need to keep some data strictly private, some data limited to defined groups, and other data shared with the wider community, have made sharing data with colleagues quite challenging. OMERO provides a permissions-based data security system for large image datasets so that sharing of defined datasets can occur at scale, over standard internet connections. OMERO implements several Permission levels, to define how data can be accessed by colleagues. Groups with different Permissions levels allow Users to control access to their data. An extension of this approach implements a Public User that provides external access to data stored in an OMERO Group. With these capabilities, OMERO enables sharing of complex, multi-dimensional image data with collaborators and, where appropriate, the wider public.
